# Muscle activation during maximal effort tasks: evidence of the selective forces that shaped the musculoskeletal system of humans

**DOI:** 10.1242/bio.014381

**Published:** 2015-11-04

**Authors:** David R. Carrier, Nadja Schilling, Christoph Anders

**Affiliations:** 1 Department of Biology, University of Utah, 257S 1400E, Salt Lake City, UT 84112, USA; 2 Friedrich-Schiller-University, Institute of Systematic Zoology and Evolutionary Biology, Erbertstr. 1, Jena 07743, Germany; 3 Clinic for Trauma, Hand and Reconstructive Surgery, Division of Motor Research, Pathophysiology and Biomechanics, Jena University Hospital, Bachstr. 18, Jena 07743, Germany

**Keywords:** Human evolution, Running, Persistence hunting, Fighting, Male-male competition, Aggression, Surface electromyography

## Abstract

The selective forces that played a role in the evolution of the musculoskeletal system of the genus *Homo* have long been debated and remain poorly understood. In this investigation, we introduce a new approach for testing alternative hypotheses. Our analysis is based on the premise that natural selection can be expected to have resulted in muscles that are large enough to achieve necessary levels of maximum performance in essential behaviors, but not larger. We used surface electromyography in male subjects to identify maximum activation levels in 13 muscles of the back and leg during eight behaviors that have been suggested to have been important to foraging, hunting and fighting performance in early humans. We asked two questions: (1) what behaviors produce maximum activation in each of the investigated muscles and (2) are there specific behaviors that elicit maximum recruitment from all or most of the muscles? We found that in eight of the 13 muscles, the highest activity occurred during maximal effort vertical jumping (i.e. whole-body acceleration). Punching produced the highest median activity in the other five muscles. Together, jumping and punching accounted for 73% of the incidences of maximum activity among all of the muscles and from all of the subjects. Thus, the size of the muscles of the back and leg appear to be more related to the demands of explosive behaviors rather than those of high speed sprinting or sustained endurance running. These results are consistent with the hypothesis that selection on aggressive behavior played an important role in the evolution of the genus *Homo*.

## Abstract (precis)

**Summary:** In this study, we identified behaviors that produce maximum activation of muscles to test hypotheses of the specific behaviors that influenced the evolution of the human musculoskeletal system.

## INTRODUCTION

Although the derived anatomical traits of the genus *Homo* are thought to have evolved in populations of hunter-gatherers (Marlowe, 2005), a consensus does not exist regarding the specific behaviors on which natural selection acted. Behaviors suggested to have played important roles in the evolution of the human postcranial musculoskeletal system include use and manufacture of tools (Napier, 1965; Marzke, 1997; Susman, 1998; Young, 2003), hunting of big game (Brace and Ryan, 1980; Frayer, 1980; Gurven and Hill, 2009), throwing (Young, 2003; Roach et al., 2013), carrying (Lovejoy, 1981; Hilton and Greaves, 2004; Wall-Scheffler et al., 2007) and economical walking (Rodman and McHenry, 1980; Lovejoy, 1981). Two additional behaviors that have more recently been added to the list are endurance running, often associated with persistence hunting, and fighting, associated with male-male physical competition. That selection for both locomotor economy and aggressive behavior may have been important in the evolution of *Homo* is intriguing because musculoskeletal characters that improve locomotor economy often are incompatible with improved performance in fighting (Pasi and Carrier, 2003; Carrier, 2004; Kemp et al., 2005; Webster et al., 2014).

**Abbreviations**

bf: musculus biceps femoris
FTE: foot extension
gc: musculus gastrocnemius
gd: musculus gluteus medius
GMX: MVC test for musculus gluteus maximus
gx: musculus gluteus maximus
HAM: hamstrings
J: squat jumping vertically
KNE: knee extension
L: lifting vertically
LGE: leg extension
LGL: leg lift
lo: musculus erector spinae, pars longissimus
M: musculus
mf: musculus multifidus
MVC: maximum voluntary contraction
P_n_: punching
P_u_: pushing laterally
R_12_: running at 12 km h^−1^
rf: musculus rectus femoris
R_max_: running at maximum speed
R_up_: running uphill at 16 km h^−1^
SEMG: surface electromyography
so: musculus soleus
SOE: Soerensen test
st: musculus semitendinosus
ta: musculus tibialis anterior
tf: musculus tensor fasciae latae
vl: musculus vastus lateralis
vm: musculus vastus medialis
W: walking at preferred speed

Modern humans are recognized as ranking among the species with the highest capacity to run long distances (Carrier, 1984; Bramble and Lieberman, 2004; Liebenberg, 2006). The exceptional performance of human endurance runners is particularly striking in light of the fact that no other primate runs long distances. Characters of *Homo* that have been suggested to have resulted from selection on endurance running include: hairlessness and a high capacity for evaporative cooling (Carrier, 1984; Lieberman, 2015); variable locomotor/ventilatory coupling patterns and relative independence between the cost of transport and running speed (Carrier, 1984); long Achilles tendons, feet with a plantar arch, large erector spinae and gluteus maximus muscles, and short toes (Bramble and Lieberman, 2004; Lieberman et al., 2006; Rolian et al., 2009).

The family of primates that includes humans, Hominidae, is characterized by relatively high levels of male-male physical competition (Harcourt, 1978; Nishida et al., 1985, 1990; Galdikas, 1985; de Waal, 1986; Goodall, 1986; Kano, 1992; Wrangham and Peterson, 1996; Furuichi, 1997; Jurmain, 1997; Wrangham, 1999; Hohmann and Fruth, 2003; Watts et al., 2006; Boesch et al., 2008; Puts, 2010; Wilson et al., 2014). This phylogenetic legacy, plus the archeological record (Keeley, 1996; Walker, 2001; Guilaine and Zammit, 2008; Estabrook, 2014; Frayer and Martin, 2014; Martin and Harrod, 2015), historical record (Keeley, 1996; Pinker, 2011), genetic diversity record (Zerjal et al., 2003; Karmin et al., 2015) and behavior of modern humans (Chagnon, 1988; Daly and Wilson, 1988; Otterbein, 2004; Puts, 2010; Wrangham and Glowacki, 2012; Sell et al., 2012; Fry and Söderberg, 2013; Walker and Bailey, 2013) are consistent with selection on aggressive behavior having been important in our evolutionary past. Given the impact of male-male and intergroup aggression on reproductive fitness in the Hominidae, it is possible that selection on aggressive behavior was one of the many factors that has influenced the musculoskeletal system of hominins. Anatomical characters that have been suggested to be associated with selection on male-male and intergroup competition include: sexual dimorphism in muscle mass, upper body strength, stature and facial robustness (Frontera et al., 1991; Puts, 2010; Carrier, 2004, 2011; Carrier and Morgan, 2015); habitual bipedalism (Darwin, 1871; Livingstone, 1962; Wescott, 1963; Jablonski and Chaplin, 1993; Carrier, 2011); and hand proportions that allow the formation of a clenched fist (Morgan and Carrier, 2012; Horns et al., 2016).

The extent to which selection on performance in running versus fighting influenced the evolution of our musculoskeletal system can be addressed, at least in part, by comparing levels of muscle recruitment necessary to perform these two behaviors. The force, work and power a muscle can produce are a function of its size; its cross-sectional area and its length. Large muscles, however, entail costs because they are expensive to maintain and transport and, in some circumstances, their inertia may actually limit rapid movements. Thus, natural selection can be expected to have resulted in muscles that are large enough to achieve necessary levels of maximum performance in essential behaviors, but not larger.

If this is true, behaviors that elicit the highest levels of activation (i.e. maximum recruitment) from a given muscle are likely among the behaviors that influenced the evolution of that muscle's size. For example, if the derived features of the musculoskeletal system of the back and hindlimb were primarily the consequence of selection for high speed running, we predict that recruitment of the various leg and back muscles would increase with running speed and be close to maximum when humans run as fast as they can. Alternatively, if selection for some other behavior, such as acceleration, was more important than maximum speed running to the fitness of early *Homo,* than we expect the locomotor muscles to be maximally activated during maximum acceleration. If, however, the musculoskeletal system of *Homo* was the result of selection for a variety of behaviors, with alternative and/or conflicting biomechanical demands, we expect that there would not be a single behavior that resulted in maximal recruitment of all or most muscles, rather a variety of different behaviors would each be associated with maximal recruitment in a small subset of the muscles of the leg and back. Thus, identification of which behaviors do and do not elicit maximum levels of recruitment can be expected to provide insight into the selective regimes that are responsible for the evolution of the musculoskeletal system of *Homo*.

In this study, we studied activation of muscles of the leg and back during several behaviors that have been suggested to have played a role in the evolution of our musculoskeletal system. Specifically, we used surface electromyography (SEMG) in adult male subjects to study walking and running at sustainable speeds, behaviors that are likely to have been important to foraging and hunting performance, and maximum acceleration, punching, pushing, lifting and sprinting, behaviors that likely influenced success in aggressive contests (i.e. fighting). We asked two questions: (1) what behaviors produce maximum activation in each of the investigated muscles and (2) are there specific behaviors that elicit maximum recruitment from all or most of the muscles? Ultimately, we hoped to improve our understanding of the selective forces that shaped the musculoskeletal system of *Homo*.

## RESULTS

**Fig. 1.**
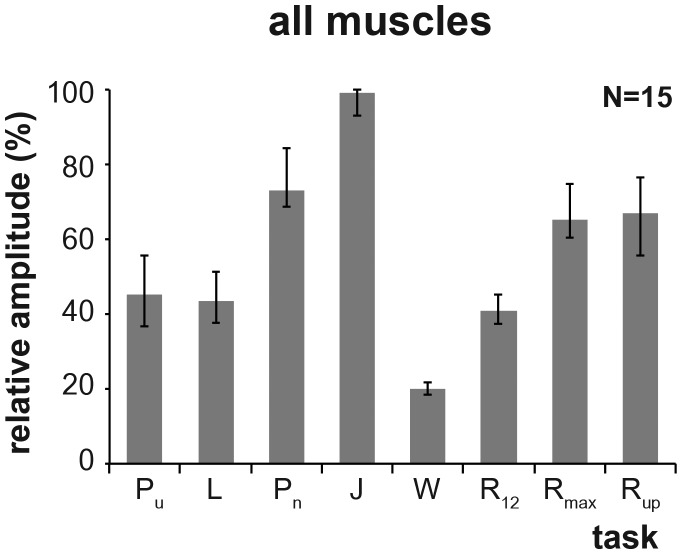
**The median activity level of the 13 muscles recorded during the eight tested behaviors.** Activity levels were measured during: pushing laterally (P_u_), lifting vertically (L), punching (P_n_), squat jumping vertically (J), walking at preferred speed (W), running at 12 km h^−1^ (R_12_), running at maximum speed (R_max_), running uphill at 16 km h^−1^ (R_up_). Error bars represent the upper and lower quartiles. Sample size of subjects is indicated in the top right corner.

**Fig. 2.**
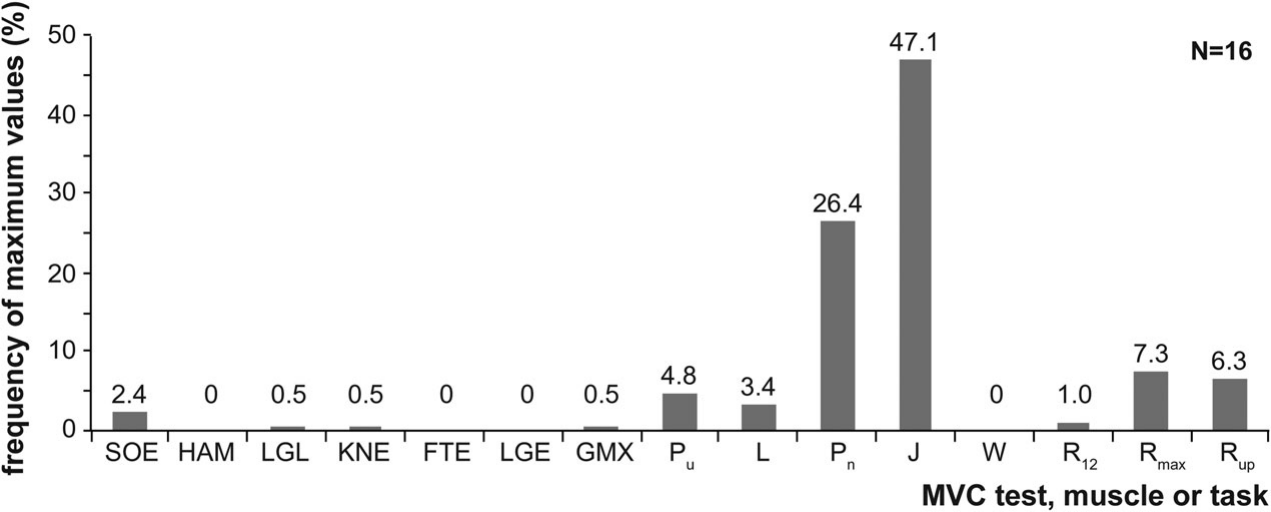
**Incidence of maximum activity from all the muscles in all of the subjects.** The first seven columns represent the incidence of maximum activity that occurred during the standard tests of maximum voluntary contraction (MVC). Sorensen test (SOE), hamstrings (HAM), leg lift (LGL), knee extension (KNE), foot extension (FTE), leg extension (LGE), musculus gluteus maximus contraction (gx). Abbreviations for the eight tested behaviors as in Figure 1.

Of the seven tested behaviors, the median activity of all 13 muscles was highest during maximal effort vertical jumping (Fig. 1). Median activity levels were intermediate during maximal effort punching, maximum speed running and running uphill at 16 km h^−1^. Running at the sustainable speed of 12 km h^−1^ and walking at the subject's preferred speed produced median activity levels that were 41% and 20% respectively of that observed during maximal effort jumping. When we asked which activity produced maximum activity in each muscle from each subject, we found that together jumping and punching accounted for 73% of the incidences of maximum activity (Fig. 2). In comparison, sprinting and running fast uphill accounted for 7% and 6% respectively of the incidences of maximum activity in any of the muscles tested. It warrants mention that the levels of individual activity in the 13 muscles was relatively low during the 7 standard maximum voluntary contraction (MVC) tests. Together, these 7 tests accounted for less than 13% of the incidences of maximum activity in any of the muscles (Fig. 2).

**Fig. 3.**
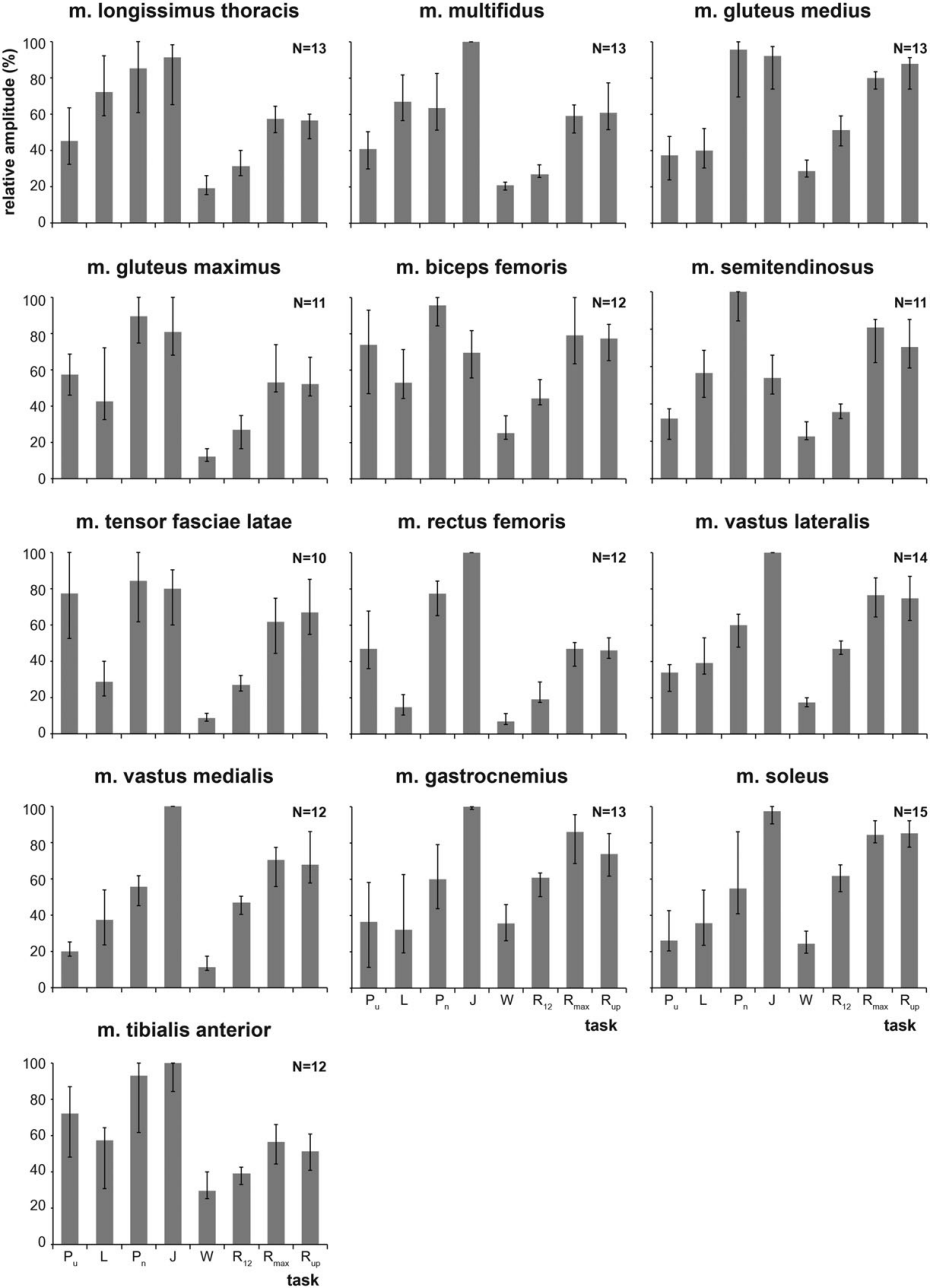
**Median peak activity observed in the different behaviors for each of the 13 muscles.** Error bars represent the upper and lower quartiles. Sample sizes of subjects are indicated in the top right corner of each graph. Abbreviations for the eight tested behaviors as in Fig. 1.

Levels of activity of the 13 muscles varied among the behaviors (Fig. 3). In 8 of the 13 muscles, the highest median activity of the subjects occurred during maximal effort vertical jumping. Punching produced the highest median activity in the other five muscles (i.e. gluteus medius, gluteus maximus, biceps femoris, semitendinosus, and tensor fasciae latae). The relative level of activity of the muscles in punching versus sprinting varied in a proximal to distal fashion. The extensor muscles of the back and the muscles that cross the hip joint exhibited greater activity in punching than in sprinting. In contrast, muscles that extend the knee and ankle tended to exhibit greater activity in sprinting than in punching.

**Fig. 4.**
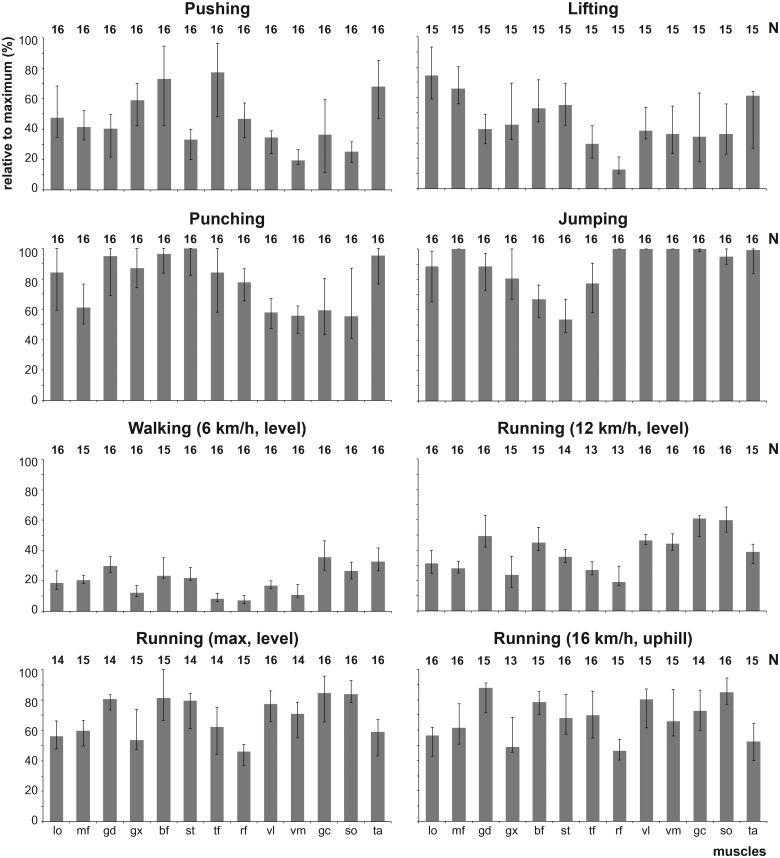
**Median peak activity observed in the 13 muscles graphed for each of the 8 behaviors to illustrate the level of variation in peak activity among the muscles.** Sample sizes for the subjects per muscle are indicated above each graph. For abbreviations of the muscles, see Table 1.

Variation in the level of recruitment among the 13 muscles also differed among behaviors (Fig. 4). The coefficient of variation was lowest for jumping (CV=21.5) followed by maximum speed running and running uphill at 16 km h^−1^ (R_max_=29.4; R_up_=29.9). Thus, the level of recruitment among the 13 muscles was more uniform during attempts at maximal acceleration or running speed than during constant speed walking and running at sustainable speed. The coefficient of variation during punching was even lower than that of sustainable speed running (P_n_=31.6, R_12_=42.8). Walking exhibited higher levels of variation (58.3), roughly equivalent to those observed during pushing and lifting (P_u_=60.4, L=54.3).

## DISCUSSION

Obviously, this study did not address all of the behaviors that may have influenced the evolution of the size of leg and back muscles. Most notably, we did not investigate spear throwing and thrusting which are both suspected to have been important to hunting success by early *Homo* (Young, 2003; Schmitt et al., 2003; Roach et al., 2013) and may also have been important in intraspecific conflict. Throwing is powered by sequential activation of many muscles starting in the legs and progressing to the trunk, shoulder and arm (Mero et al., 1994; Hirashima et al., 2002). A recent analysis has shown that our ability to throw at high velocity results largely from several derived anatomical features that enable storage and recovery of elastic strain energy at the shoulder joint (Roach et al., 2013). Specialization of the musculoskeletal system for throwing may extend beyond the shoulder to the back and legs. Spear thrusting, which may have preceded throwing as a hunting method, involves different patterns of muscle recruitment, and requires close quarter interaction with potentially dangerous prey animals (Schmitt et al., 2003). Carrying is another behavior that can be taxing and has been suggested to have been important in the evolution of *Homo*, often in the context of provisioning of offspring (Isaac, 1978; Lovejoy, 1981; Hill, 1982; O'Connell et al., 1999; Hilton and Greaves, 2004; Wall-Scheffler et al., 2007). Carrying heavy loads as might occur after a successful hunt or when a female carries both an infant and a load of food or firewood likely requires higher levels of muscle activation and the required effort would increase when walking uphill or in loose sand (Lejeune et al., 1998). By contrast, carrying loads can be managed with relatively high mechanical advantage if the respective load is carried with low lever arms, i.e. close to the body or even on the head (Griffin et al., 2003). The latter technique can still be seen in African nomadic hunter-gatherer people (Maloiy et al., 1986). Nevertheless, this study would have been strengthened by the inclusion of throwing, thrusting, and carrying among the tested behaviors.

The results of this study are consistent with the hypothesis that the size of the muscles of the back and leg are more a function of selection for explosive behaviors than sustained endurance running. The median activity of the 13 muscles was highest during maximal effort vertical jumping. Together jumping and punching accounted for 73% of the incidences of maximum activity among all of the muscles and from all of the subjects. In eight of the 13 muscles, the highest median activity of the subjects occurred during maximal effort vertical jumping. Punching produced the highest median activity in the other 5 muscles. Finally, the coefficient of variation was lowest for maximal effort jumping. These results are consistent with the hypothesis that selection for interspecific aggression has played an important role in the evolution of our musculoskeletal system.

For many of the 13 muscles, activity during maximal effort sprinting ranked second or third behind maximal effort jumping or punching. Nevertheless, because the maximum running speeds of humans are much lower than those of most prey and predator species (Garland, 1983), we suspect that sprinting would rarely have been useful in a hunting context, in avoiding attack from predators, or in racing predators to the site of a recently killed carcass. Sprinting during hunting might be important to scare prey toward an ambush or to close the distance to facilitate a spear throw. But these may have been rare opportunities in most environments. In contrast, maximum running speed may have often been a factor in determining the outcome of intraspecific aggression. Thus, we suspect that sprinting in early *Homo* was a behavior that was more likely to have been important during intraspecific aggression than during interactions with other species.

For each of the behaviors we studied, there was one or more muscle/s that exhibited relatively low levels of recruitment and can therefore be viewed as being “overbuilt” for that behavior. Although maximal effort jumping produced high levels of recruitment from all of the muscles investigated, the hamstring muscles were recruited at low levels relative to the other muscles. Muscles that are overbuilt for sprinting are the epaxials, gluteus maximus, rectus femoris, and the tibialis anterior. Muscles that are overbuilt for maximal effort punching are the extensors of the knee and ankle. Interestingly, all of the muscles exhibited relatively low levels of recruitment during maximal effort lateral pushing and vertical lifting. This might represent a mechanism to protect the integrity of the back and leg from potentially damaging rotational and vertical moments.

As anticipated, the muscles of the back and leg exhibited low to intermediate levels of activity during walking and running at sustainable speeds. This result is consistent with previous studies that have compared activity of leg muscles during running at intermediate speeds to sprinting (Kyröläinen et al., 2005; Bartlett et al., 2014). These results are not surprising given that muscle moments (Roberts and Belliveau, 2005) and forces applied to the ground (Kram and Taylor, 1990) increase with walking and running speed and are generally low during walking and running on level ground at speeds that can be sustained. Moreover, these observations are not consistent with the suggestion that the size of some of the extensor muscles of the back and leg of humans are a result of selection for endurance running capacities (Bramble and Lieberman, 2004; Lieberman et al., 2006). Compared to running at speeds that can be sustained, the epaxial and gluteus maximus muscles exhibit substantially higher levels of activity during sprinting (Bartlett et al., 2014; this study) and substantially higher activity during maximal effort jumping and/or punching than during sprinting. If the musculoskeletal system of humans was solely or primarily specialized for economical long distance running, the size and gearing of our muscles would restrict our fastest running speeds to values that were not much greater than our maximum sustainable speeds. Although humans are economical walkers (Rodman and McHenry, 1980; Cunningham et al., 2010) and rank among the elite species when running long distances, the results of this study suggest that our musculoskeletal system is also specialized for aggressive behavior.

Although the extent to which the muscles of extinct hominin species were specialized for locomotor or fighting performance cannot be directly demonstrated, the fossil record does provide relevant clues. Several of the skeletal characters most diagnostic of the hominin lineage have been suggested to be functionally consistent with anatomical specialization for aggressive behavior, including habitual bipedal posture (Darwin, 1871; Livingstone, 1962; Wescott, 1963; Jablonski and Chaplin, 1993; Carrier, 2011), proportions of the hand that allow formation of a fist (Morgan and Carrier, 2012; Horns et al., 2016), robusticity of the facial skeleton (Carrier and Morgan, 2015), sexual dimorphism of the upper body and arms (Frontera et al., 1991; Puts, 2010; Carrier, 2004, 2011), and sexual dimorphism of the facial skeleton (Carrier and Morgan, 2015). These anatomical correlates combined with the importance of physical aggression in the male-male and intergroup competition of great apes, including humans (see references cited above), suggests that patterns of muscle activation we observed in modern humans are likely to be applicable to species of early *Homo*. In contrast to the derived skeletal traits of basal hominins, many of the distinguishing characters of the post cranial skeleton of the genus *Homo* appear to be more consistent with specialization for economical walking and running rather than specialization for aggressive behavior, including the increased length of the legs (Bramble and Lieberman, 2004; Sockol et al., 2007; Steudel-Numbers et al., 2007), reduced length of the toes (Rolian et al., 2009), and reduced upper body mass and strength (Bramble and Lieberman, 2004). Given the functional tradeoffs inherent in specialization for economical locomotion versus specialization for fighting, the increased gracility of the postcranial skeleton could be interpreted as evidence that aggressive behavior was of relatively low importance to the fitness in species of early *Homo*. However, among extant mammals, modern humans are a relatively violent species and the archeological and genetic evidence suggest that violent behavior is deeply rooted in our evolutionary history (references cited above). In this context, the gracilization of the postcranial skeleton in *Homo* may represent changes in the manner of physical combat that were associated with the invention of effective weapons; weapons that reduced constraints on specialization for economical locomotion and allowed aggressive behavior that was less dependent on physical strength (Carrier, 2004).

## MATERIALS AND METHODS

We investigated 17 healthy, recreational active males (age: 31.8±8 years, height: 1.79±0.05 m, mass: 73.7±7.2 kg, BMI: 22.9±1.5 kg m^−2^, data given as mean±standard deviation) with no history or presence of orthopaedic and cardiovascular diseases. All gave their informed consent to voluntarily participate in this study, which was approved by the ethics committee of the University Hospital Jena, Germany (0558-11/00).

We simultaneously recorded surface EMG from 13 different muscles of the back and legs bilaterally (muscles and electrode positions are detailed in Table 1) in accordance with international recommendations (Hermens et al., 1999). After gentle cleaning and shaving of the skin, disposable Ag-AgCl electrodes (H93SG, Arbo, Neustadt, Germany) with a circular uptake area of 1.6 cm diameter and an inter-electrode distance of 2.5 cm were applied. All electrodes and amplifiers were carefully secured with tape and elastic net bandages to minimize movement artefacts. Data were amplified (gain: 1000, biovision, Werheim, Germany) and stored on a computer (GJB Datentechnik, Langewiesen, Germany; AD-conversion at 2000 s^−1^ DAQ-Card-AI-16E-4, 12 bit, National Instruments, Austin, TX, USA) for analysis.

**Table 1.**
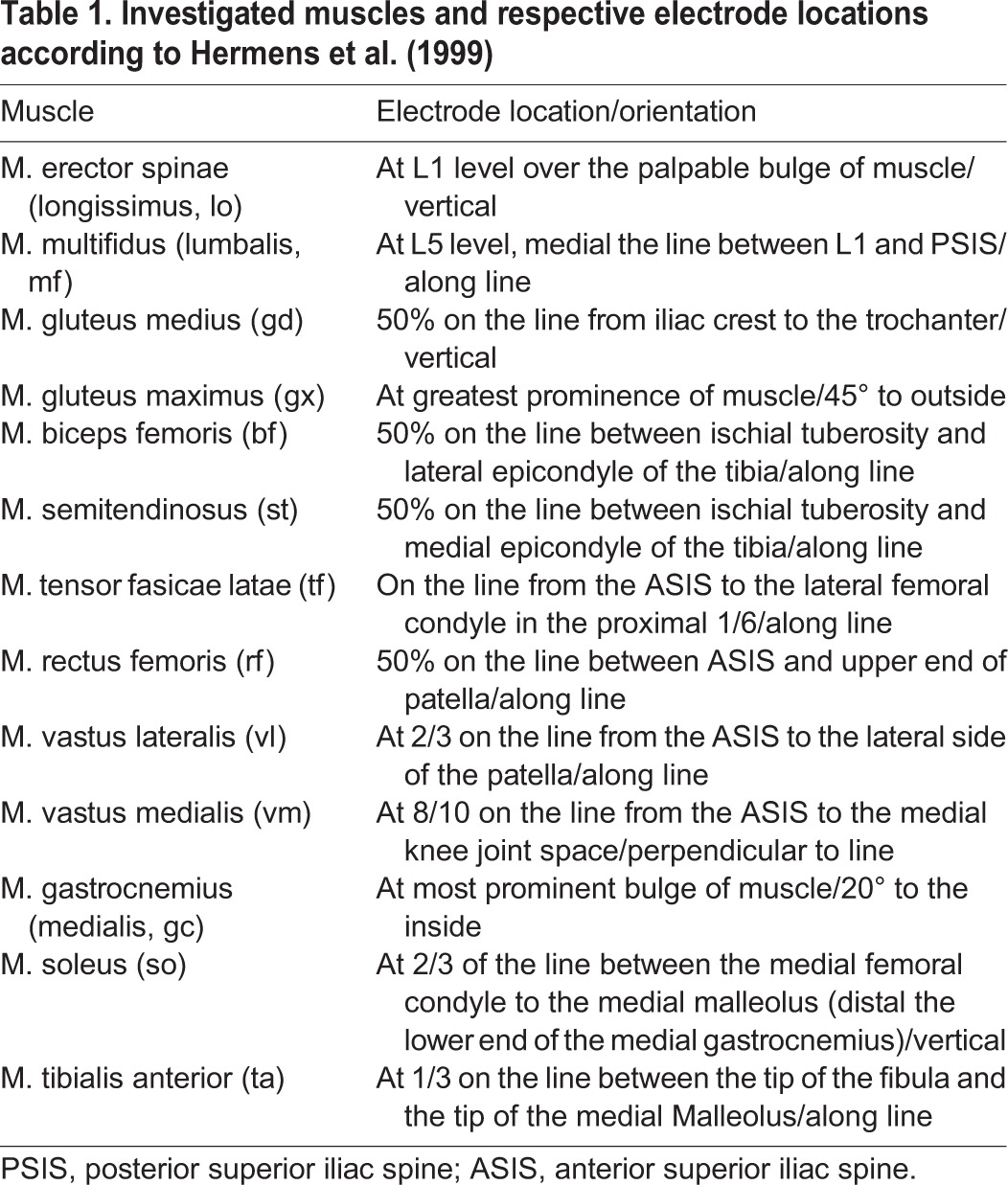
**Investigated muscles and respective electrode locations according to** **Hermens et al. (1999)**

During testing, the subjects completed seven different standardized tests of maximum voluntary contraction (MVC) and eight different whole body behaviors; four of which were high force or power behaviors and the other four were locomotor behaviors. The standardized MVC tests were applied according to Kendall et al. (2005) to evoke maximum efforts of the investigated muscles and were performed before the locomotor tests. They were all performed three times as was done also for the whole body behaviors. To minimize fatigue, only 20 strides were recorded per subject and locomotor task. Because all subjects were experienced and regular treadmill users, they rested by standing on both sides of the treadmill with their legs spread apart while the treadmill speed was adjusted. Therefore, they completed only approximately 20 strides per task. We used this particular procedure to account for possible muscle fatigue effects and, at the same time, to comply with the demands of gait analysis, which requires about 15 complete strides (Winter and Yack, 1987). The whole body behaviors were performed after the locomotor tasks.

In the *Squat Jumps*, test subjects completed maximal effort vertical jumps in quick succession. This test provided an indication of muscle activity during maximal attempts at whole body acceleration. To determine the levels of muscle recruitment during a grappling (*Lateral Push*), subjects pushed laterally with both hands, perpendicular to the direction they faced, as hard as possible, for several seconds first to the left and then to the right against a doorframe. To monitor activity levels during maximal effort *Vertical Lifting*, subjects attempted to lift a waist high counter top secured to the wall of the laboratory. In the *Punching* test, subjects wore boxing gloves and struck a 45 kg punching bag as hard as they could with forward boxing style punches. To determine the muscle activity during economical *Walking*, subjects walked on a treadmill at their preferred (self-chosen) walking speed. Muscle activity during *Endurance Running* was recorded as the subjects ran on a treadmill at a speed of 12 km h^−1^. This speed represented a comfortable and sustainable speed for all of our subjects and is somewhat greater than the average running speed of Kung Bushmen hunters recorded during successful persistence hunts (Liebenberg, 2006). The muscle activity during *Maximum Speed Running* was recorded by having the subjects run on the treadmill at the speed that they could just barely maintain for 20 strides. This speed varied from 18 km h^−1^ (one subject) to the maximum running speed of 22 km h^−1^ (10 subjects). To determine the level of activity during *Running Uphill*, the subjects ran on a treadmill inclined at 10° and at a speed of 16 km h^−1^. During the walking and running trials, the left foot was equipped with an accelerometer to accurately identify the beginning of a stride, defined as the moment of contact of the left foot with the surface of the treadmill.

All analyses were performed by custom-made programs using MATLAB (The MathWorks, Natick, MA, USA). For locomotion analysis, EMGs were sampled and analyzed on a stride-by-stride basis, resulting in grand averaged, time normalized curves for every single muscle, speed and subject, respectively. The exact procedure is detailed elsewhere (Carrier et al., 2011).

To determine maximum muscle activation, we chose to quantify the maximum voltage value recorded during the trial. We visually identified the EMG signal associated with each trial and recorded the highest absolute voltage value. The maximum muscle activity levels of the standardized tests were determined by visual inspection of all trials by the same investigator (DRC) to ensure stable ratings. Maximum voltage values for the different behaviors were normalized relative to the behavior for each subject that exhibited the highest voltage value. Thus, the normalizing behavior varied among subjects and among muscles. Graphical displays of the results are presented as median values±quartile ranges because the median is less susceptible to outliers and also robust against non-normally distributed data.

Due to the extremely strenuous and partly explosive task requirements, subjects tended to perspire at a high level during the recording session. This sometimes resulted in detachment of one or more of the electrodes. All signals were carefully monitored during the measurements, but due to the strenuous requirements of some tasks, trials could not be repeated, even if detached electrodes were identified. These corrupted signals were excluded from the analysis. Therefore, valid sample size varies between tasks and muscles. The respective sample sizes are provided in every graph.
